# Metacarpal reconstruction with a medial femoral condyle flap based on a 3D-printed model: a case report

**DOI:** 10.1080/23320885.2022.2029453

**Published:** 2022-01-21

**Authors:** Manfred Schmidt, Matthias Holzbauer, Stefan M. Froschauer

**Affiliations:** a Section of Plastic and Reconstructive Surgery, Kepler University Hospital, Linz, Austria; b Medical Faculty, Johannes Kepler University Linz, Linz, Austria; c Clinic for Orthopedics and Traumatology, Kepler University Hospital, Linz, Austria

**Keywords:** 3D printing, metacarpal, giant cell tumor, reconstruction, medial femoral condyle flap

## Abstract

Metacarpal bone reconstruction renders a surgical challenge. We describe a case using 3D printing assisted medial femoral condyle flap for extensive metacarpal reconstruction after wide resection of a large giant cell tumor recurrence. Thus, the length and stability of the entire third ray could be restored without any tumor recurrence. (50 w)

## Introduction

Giant cell tumors (GCT) of bone represent approximately 5% of primary bone tumors [1], while the bony skeleton of the hand is rarely affected by this tumor entity. Metacarpals, followed by phalanges, are reported to be the most common site of GCT of bone in the hand accounting for 2−5% of all GCT of bone with a peak of occurrence in the fourth decade of life [2–4]. Despite being considered benign, GCT may progress aggressively and lead to considerable local bone destruction and functional impairment or even pulmonary metastases in 1–4% [1]. Thus, the eradication of the disease is the primary therapeutic goal, while secondary considerations are given to functional reconstruction and aesthetic integrity of the hand. These principles for surgical treatment of GCT of bone pose a reconstructive challenge, especially for repair of defects encompassing the majority of a metacarpal bone. Medial femoral condyle (MFC) flap has emerged as a versatile reconstructive option for a variety of small to medium sized bone defects of the hand [5]. It has also been reported for partial metacarpal reconstruction [6]. Three-dimensional (3D) printed models have recently been described as an innovative technology for facilitating MFC flap harvest and for precise tailoring of the required bone flap [7].

We describe a case using 3 D printing assisted medial femoral condyle flap for extensive metacarpal reconstruction after resection of a large giant cell tumor recurrence of the third metacarpal bone.

## Case report

**Figure 1.**
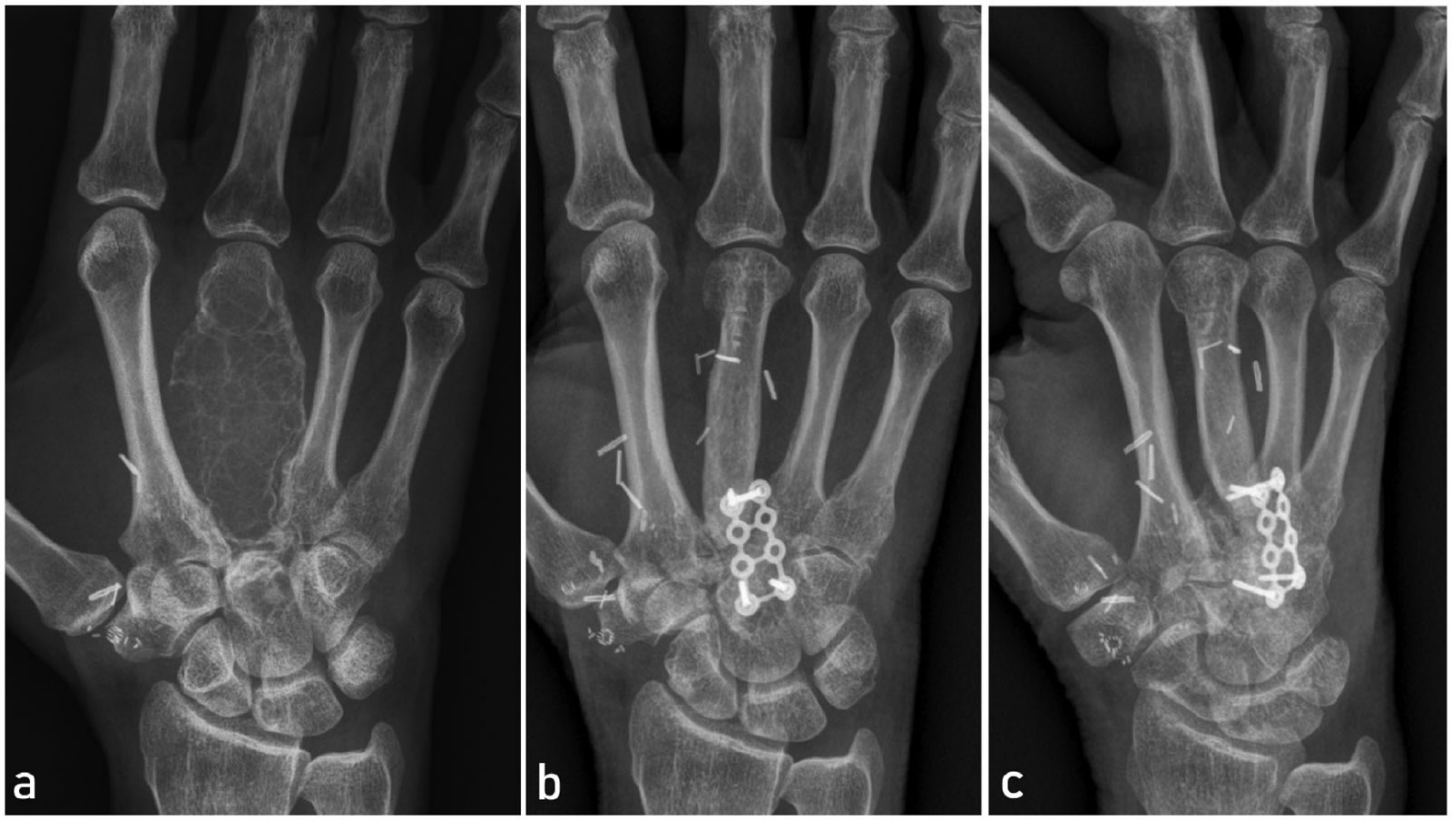
Preoperative x-ray showing the tumor encompassing the entire third right metacarpal including the metacarpal head (a). Follow-up x-rays one year after metacarpal reconstruction with MFC bone flap in dorsopalmar (b) and lateral view (c). Note the preservation of metacarpal length and distal bone remodeling at the metacarpal head.

A 47-year-old woman presented with an enlarging soft tissue mass at the dorsum of her right hand, two years after resection of a giant cell tumor (GCT) of her third metacarpal at another hospital. We performed wide excision of the tumor and covered the defect with a free suprafascial anterolateral thigh flap. Histological analysis revealed a rapidly growing GCT recurrence. Three-and-a-half years later, plain radiographs and a CT-scan revealed local tumor recurrence. The tumor involved the entire metacarpal base, the shaft and more than half of the metacarpal head (Figure 1(a)). The lesion measured 5.3 cm in diameter and reduced the metacarpal length by 5 mm. Preoperatively, the range of motion of the third metacarpophalangeal joint was limited to 10° of active flexion due to adhesions of the extensor digitorum tendon. CT-scans of the affected third metacarpal were performed (Siemens Healthineers, Erlangen, Germany). These data were further processed using a 3D simulation software (3-matic, version 12.0.0, Materialise, USA). A hand surgeon and a computer technician have virtually planned a model of the corticocancellous flap, which would perfectly fit for metacarpal reconstruction (see Supplementary Video 1). At the future entry point of the vascular pedicle, which was chosen according to the relatively constant vascular anatomy of the MFC, a handlebar was added. Following, this model was printed with a Formlabs 2 printer (Formlabs Inc, Massachusetts, USA) with a clear photopolymer resin (Form 2 dental SG cartridge, Formlabs Inc, USA), cleaned in isopropanolol and hardened at 62 °C. Preoperative preparation was finished via sterilizing and packing this template for intraoperative use.

Surgical procedure started *via* partially elevating the covering flap and resecting the entire tumorous third metacarpal except for the subchondral part of the head.

The sterilized template was afterwards used to examine the fit of the planned flap size while placing the model into the metacarpal defect. Thus, we were able to control the metacarpal length and plan the miniplates used for osteosynthesis.

**Figure 2.**
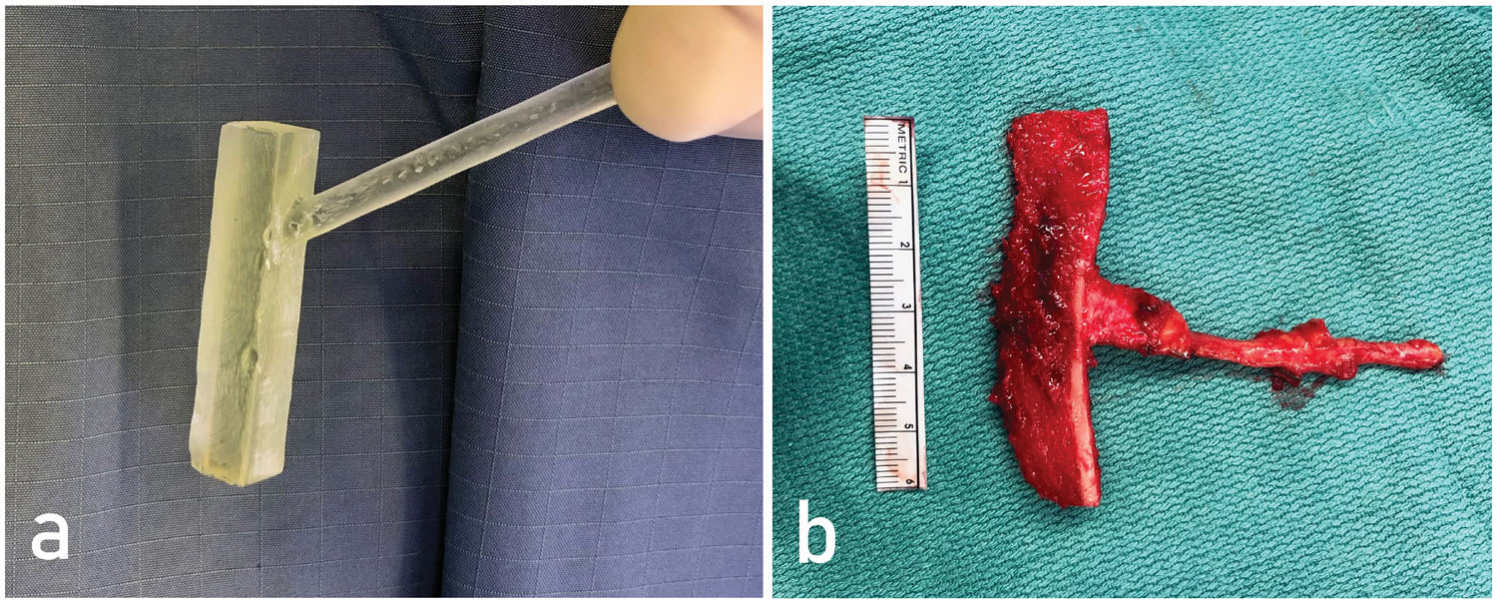
A sterile 3D printed model with a handlebar virtually designed to perfectly fit in the metacarpal defect after tumor resection was used for bone flap elevation. Corticocancellous MFC bone flap after elevation (b).

If the size of the future free corticocancellous flap was verified, the same template was used to determine most suitable harvest site for a microvascular medial femoral condyle (MFC) flap. Moreover, resection line was directly marked by copying the contour of the 3D model. After harvesting the free MFC flap via an oscillating saw and chisels, the size of the 3D template and the flap perfectly matched (Figure 2). The corticocancellous vascularized flap was fixated in the metacarpal defect with two 2 mm fixed-angle locking plates. The flap was connected to the radial artery via an end-to-side anastomosis and an accompanying vein using a vein coupler. The procedure also contained extensive tenolysis of the extensor digitorum tendon and intraoperative mobilization of the metacarpophalangeal joint. Postoperative regimen included a thermoplastic splint for 6 weeks. Hand therapy was started after wound healing was finished and continued 6 months postoperatively. The normal length of the third metacarpal was reconstructed and bony union was obtained.

**Figure 3.**
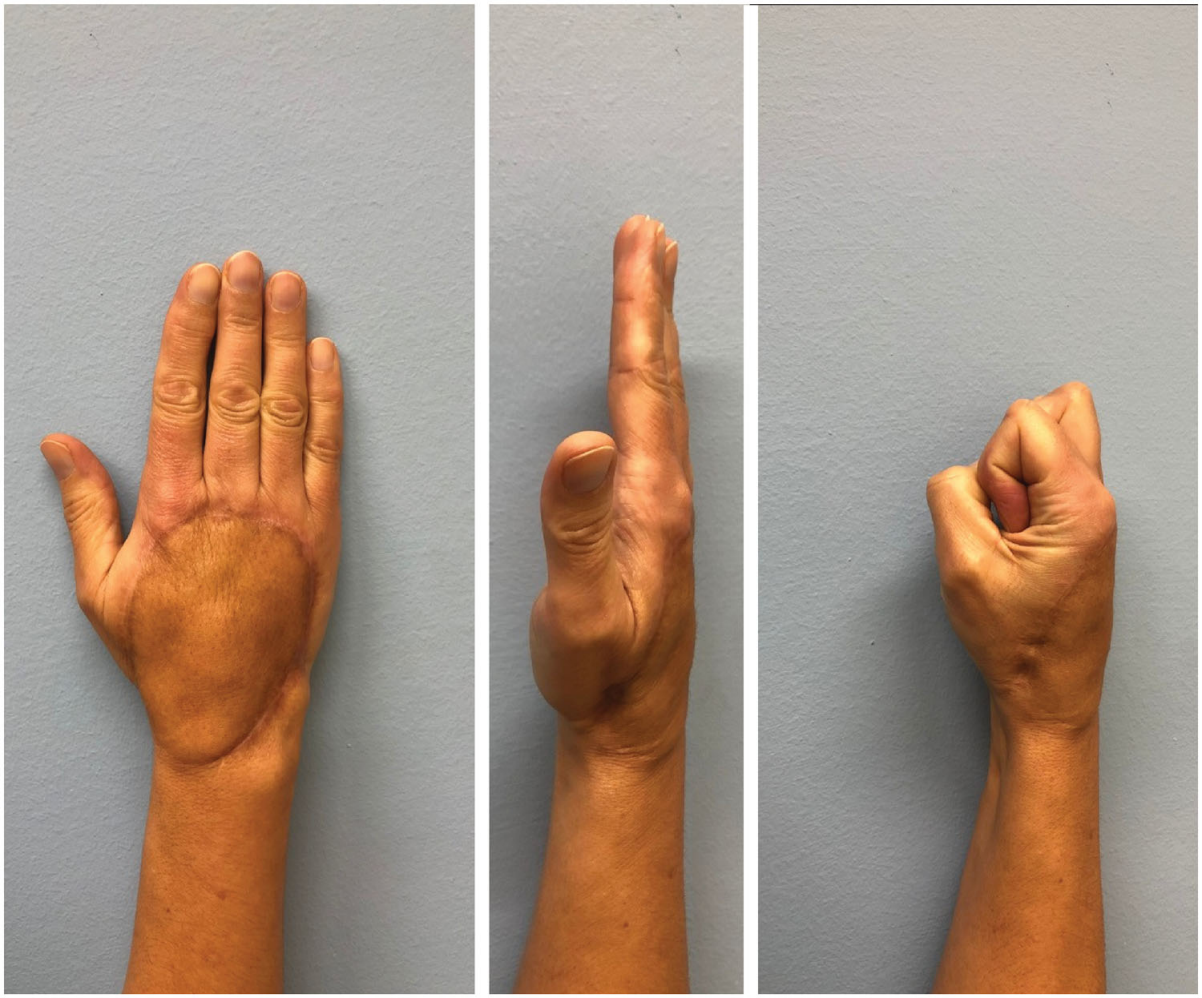
Photograph of the affected hand in two planes two years postoperatively.

One year after surgery, the locking plates were removed. Proximally, a protruding ossification of the bone flap was trimmed and a bone cyst at the capitate was debrided, filled with cancellous bone flap and the proximal MFC flap was fixed to the capitate with a mini-plate (Figure 1(b,c)). There was no histological evidence of tumor recurrence. At two years follow-up, there was no clinical or radiographic sign of tumor recurrence as well as no donor site morbidity. Moreover, the final metacarpal length was improved compared to the preoperative one. However, we unfortunately observed some subsidence of metacarpal length during follow-up and range of motion could only be improved to 30° flexion in the metacarpophalangeal joint. The range of motion of the proximal and distal interphalangeal joint was not impaired. While this restriction did not represent a subjective functional disability of the hand function for our patient, this procedure preserved the aesthetic integrity of the hand (Figure 3).

## Discussion

Diagnosis of GCT of bone – especially in early stages – renders a difficult task because patient’s symptoms are unspecific ranging from pain, local swelling, or limited range of motion of the adjacent joint [4], while pathologic fractures due to substantial cortical destruction lead to initial presentation in 15−20% of patients [4]. Radiographcially, this tumor entity appears as simple, eccentric lytic lesion in stage 1, evolving to a formation with no radiopaque rim and thinned cortex (stage 2) and finally leads to a lesion with indistinct boarders and cortical destruction (stage 3) according to classification proposed by Campanacci et al. [8].

Surgical treatment represents the gold standard in the management of GCT of bone. However, the individual risk of local tumor recurrence related to various surgical methods has to be considered. Thus, the more radical a GCT lesion is resected, the lower the rate of local recurrence, however, the higher the reconstructive challenge. While simple curettage leads to a recurrence rate of 27−65% [4], which can be reduced to 12−27% [4] using adjuvants, en-bloc, wide resection remains the only alternative for primary treatment of an advanced stage or tumor recurrence treatment – as it is the case in the current patient.

Surgical procedures basically involve ray resection as well as metacarpal reconstruction using various techniques. The former allows an extended resection, which is especially important in tumor entities with aggressive infiltration; hence, a low recurrence rate can be achieved, if tumor-free excision margins can be achieved during initial surgery [9]. Moreover, ray resection leads to immediate, good functional results with low complication rates if negative margins (R0) were accomplished. However, after extensive patient information, preserving the entire aesthetic integrity of the hand was of utmost importance for our patient. Thus, we were considering current methods for metacarpal reconstruction: the commonly applied techniques involve both non-vascularized and vascularized autologous bone flaps from the fibula, the iliac crest or the distal radius for smaller defects [10]. Recently, MFC represents an increasingly utilized donor site, where free vascularized osteochondral [7] or corticocancellous microvascular bone flap up to a maximal length of 13.7 cm [11] can be harvested.

For extensive metacarpal reconstruction the free vascularized MFC corticocancellous bone flap implies several benefits. In contrast, alternative bone flaps such as derived from the iliac crest are too curved and the fibula is too massive. Albeit the MFC flap consist of a single layer of compact bone, the cortical thickness of the MFC warrants a large contact surface for bony union, hence, postoperative stability for the reconstructed finger ray. In contrast, the tricortical properties of the iliac crest and the triangular shape of the fibula commonly entail the need for excessive, intricate flap trimming, which renders osteosynthesis even more challenging. Moreover, the MFC could serve as an osteochondral flap to reconstruct a metacarpal lesion encompassing the total metacarpal head including the proximal joint surface of the metacarpophalangeal joint.

Recently, haptic, 3 D-printed models on the basis of CT data have emerged as novel, intraoperative assistant devices improving the surgeons’ 3D perception while performing intricate, surgical procedures [7]. In our particular case, 3D models were used for three purposes: First, the model enabled a functional verification of the flap size while placing it into the metacarpal defect, even before the final MFC flap dissection, harvesting etc. had even been started. Secondly, it assists the surgeon via identifying the most suitable donor site. Thus, the MFC can be screened for the location where the curvature of cortical surface and the exact vascular anatomy fit most for metacarpal reconstruction. Although the surgeon also considers the approximate size of the planned flap for choosing the harvest site during free-hand flap harvesting, however, a 3D template enables a constant, optical comparison between the desired flap shape and the definite MFC’s surface and vascular supply. Finally, the resection line can directly be marked by copying the contour of the 3D model, which renders arduous measuring of each resection line redundant. Following, the flap size can be chosen extremely accurately. This step reduces donor site morbidity, and leads to better functional outcome. Furthermore, this step also minimizes the consequent need for flap trimming, which conventionally often leads to inaccurate results and increases the ischemia time of the flap [7].

In the present case, a wide excision of a recurrent GCT lesion of nearly the entire metacarpal was performed causing a large bony defect. For reconstruction a perfectly fitting, corticocancellous microvascular bone flap of the MFC was performed, facilitated by the intraoperative use of a 3D printed model. Finally, this procedure yielded a stable reconstruction of the third metacarpal with restored length of the third ray. The aesthetic integrity of the overall hand could be preserved, and most importantly, the patient remained recurrence-free for the entire follow-up period of one year. (1589 W)

## Supplementary Material

Supplemental Material
